# Beyond the gene: isoform diversity as a key contributor to human brain disorders^✰^

**DOI:** 10.1016/j.gde.2026.102497

**Published:** 2026-06-09

**Authors:** Yunlong Ma, Connor Jops, Michael J Gandal

**Affiliations:** 1 Departments of Psychiatry, Genetics, and Pediatrics, Perelman School of Medicine, University of Pennsylvania, Philadelphia, PA, USA; 2 Lurie Autism Institute at Penn Med and the Children’s Hospital of Philadelphia, Philadelphia, PA, USA

## Abstract

The human brain exhibits exceptional transcriptomic complexity, with alternative splicing, promoter usage, and polyadenylation generating extensive transcript-isoform diversity. Isoform dysregulation is increasingly implicated in neurodevelopmental and psychiatric disorders (NPDs), yet the landscape, function, and genetic regulation of brain isoforms remain poorly understood due to limitations of short-read RNA sequencing. Advances in long-read sequencing (LR-seq) enable scalable full-length transcriptome profiling with single-cell and spatial resolution across developmental stages. Here, we review recent progress in isoform discovery, quantification, functional annotation, and genetic regulation, highlighting emerging links to human neurodevelopment and disease. LR-seq studies have uncovered tens of thousands of previously unannotated brain isoforms, with neuronal maturation characterized by increased exon inclusion and progressive 3′ untranslated region (3′ UTR) lengthening. Isoform-resolved genetic mapping outperforms gene-level analyses for NPD gene discovery and mechanistic interpretation. We argue that a shift from gene-centric to isoform-centric frameworks is essential to fully capture regulatory complexity in human neurogenetics. Together, these advances establish isoform diversity as a fundamental yet underappreciated axis of brain gene regulation and a key entry point for dissecting NPD biology.

## Introduction

**Figure 1:**
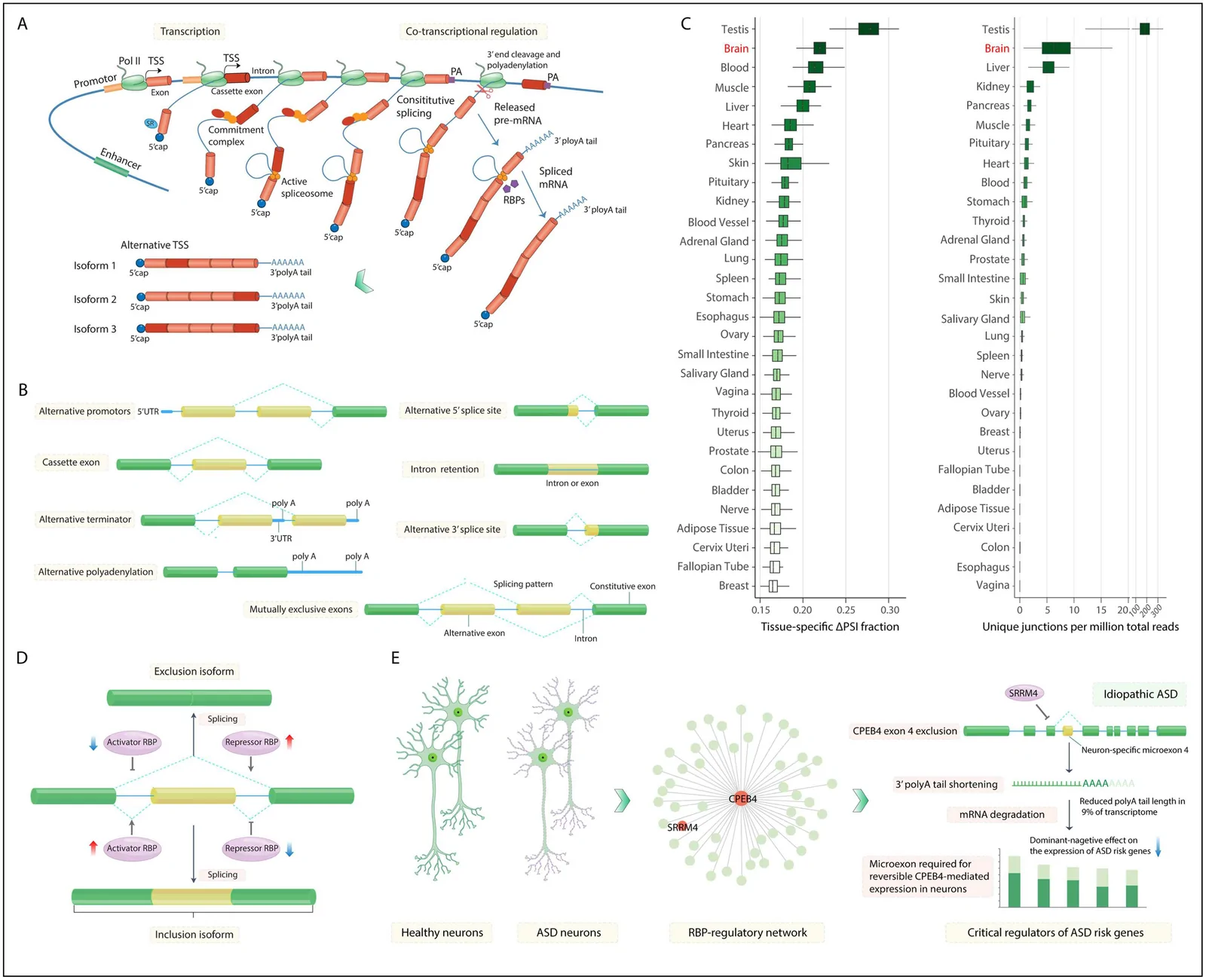
Tissue-wide patterns of isoform diversity and neuron-specific regulation. **(a)** Co-transcriptional regulation of alternative splicing and isoform diversity. RNA polymerase II (Pol II) initiates transcription at alternative transcription start sites (TSSs) within promoter regions, producing pre-mRNAs containing constitutive and cassette exons. During transcription elongation, the commitment complex and active spliceosome assemble in a co-transcriptional manner to mediate constitutive and alternative splicing. In parallel, regulated 3′-end cleavage and alternative polyadenylation (APA) determine transcript termination and poly(A) site usage. RNA-binding proteins (RBPs) further modulate splicing and 3′-end processing. The coordinated regulation of alternative TSS selection, splicing, and APA generates multiple mature mRNA isoforms with distinct 5′ and 3′ ends and internal exon compositions. **(b)** A schematic representation of alternative pre-mRNA splicing in eukaryotic genes, illustrates multiple distinct transcript-level effects: alternative 5′ splice site, alternative 3′ splice site, intron retention, cassette exon, alternative terminator/3′UTR, alternative polyadenylation, alternative promoters, and mutually exclusive exons. Constitutive exons are shown in green; alternative exons or exon fragments are in yellow. **(c)** Cross-tissue quantification of splicing diversity. Left: Tissue-specific ΔPSI fraction. For each sample, we computed the fraction of *detectable* splicing events whose Percent Spliced-In (PSI) differs from the median PSI of all other tissues by at least |ΔPSI| ≥0.20; ‘detectable’ requires non-missing PSI in both groups with ≥ 10 samples per side. PSI values were quantified with SUPPA2 [56] across canonical event classes. Right: Unique junctions per million total reads. For each tissue, we first defined a repertoire of tissue-present splice junctions (CPM ≥ 0.1 in ≥ 20% of samples or at least 10 samples). Junctions were called tissue-unique if they were present in that tissue but absent in all others (< 2% of samples). For the brain, presence/absence was evaluated at the subregion level and then OR-combined to obtain the brain repertoire. For each sample, we then counted how many of that tissue’s unique junctions were expressed (CPM ≥ 0.1) and normalized by the sample’s total mapped reads (per million). Boxplots summarize the per-sample values; an axis break is used to accommodate the high values in the testis. The brain is highlighted in red. Data: GTEx v10 RNA-seq across human tissues [57]; exon–exon junction counts from STAR; per-sample normalization by total read depth. Values are shown as boxplots grouped by tissue (center line = median; box = IQR; whiskers = 1.5×IQR). **(d)** Conceptual model of RBP control of exon choice in a context-dependent, temporal, and spatial manner. Recruitment of RBPs that act as activators or repressors at flanking splice sites shifts spliceosome usage, producing inclusion or exclusion isoforms. **(e)** Example of neuronal RBP network and ASD-linked perturbation. Cartoon summary of a neuron-specific regulatory network in which RBPs (e.g. SRRM4 and CPEB4) coordinate microexon inclusion.

Following the central dogma of biology, genetic information flows from DNA to RNA to protein. For the approximately 20 000 protein-coding genes in the human genome, gene expression begins with transcription factor binding at enhancer and promoter regulatory elements, which recruits RNA polymerase II to begin transcription of DNA into precursor messenger RNAs (pre-mRNAs). As pre-mRNAs are elongated, they undergo extensive post/co-transcriptional RNA processing [1]. Most notably, this includes 5’ mRNA capping, alternative splicing (AS) of intronic sequences, and alternative polyadenylation (APA) via poly-A site selection and polyA tail ligation (Figure 1a). Originally thought to occur sequentially, it is now widely appreciated that transcription and RNA processing are highly coordinated and concurrent. This intricate process, governed by machinery including RNA-binding proteins (RBPs) and the spliceosomal complex, among others, results in a diverse set of mRNA transcript-isoforms emanating from the same genomic locus (Figure 1b). Distinct mRNA isoforms produced from the same gene can differ in at least four functionally consequential ways: they may encode distinct protein isoforms with divergent, and in some cases even antagonistic, biological activities; they have undergone non-sense mediated decay; they may localize to different subcellular compartments, such as the nucleus, cytoplasm, or synapse; or undergo distinct post-transcriptional regulation, for example through differential microRNA or RBP-based targeting [2,3]. Despite this molecular diversity, conventional genetic and transcriptomic analyses typically regard the ‘gene’ as the final common product of a given genomic locus, collapsing isoforms into aggregate gene expression, thereby obscuring isoform-specific regulation [1]. Recent advances in long-read sequencing technologies and isoform-centric quantification are beginning to shift this paradigm.

Over 95% of multiexon genes are alternatively spliced, expanding coding and regulatory potential. Across all human tissues, the brain exhibits the most extensive patterns of AS and resulting isoform diversity (Figure 1c), a feature thought to support the evolution of higher-order human cognitive functions. Brain-expressed genes are longer, contain more exons, and undergo highly complex splicing regulation, which contributes to the evolutionary and phenotypic complexity of the human brain [4–7]. Synaptic genes, in particular, often show striking isoform diversity and are strongly implicated in neurodevelopmental disorders (NDDs). For example, the presynaptic cell adhesion module and NDD risk gene *NRXN1* have more than 1000 unique isoforms. Isoforms are highly context-dependent across regions, cortical layers, and developmental stages [8–10], with tightly regulated AS governing neuronal identity, connectivity, plasticity, and migration (Figure 1d). Microexons — short exons of 3–27 nucleotides — represent a unique class of neuronal splicing events that are highly evolutionarily conserved and exhibit switch-like regulation. These microexons often modulate protein interaction domains involved in neurogenesis and play critical roles in neuronal differentiation and synaptic function. Dysregulation of neural microexons has been frequently observed in the brains of individuals with autism spectrum disorder (ASD) [11], highlighting their functional and disease relevance (Figure 1e).

It is now widely established that the human brain expresses the longest 3’ untranslated regions (3′ UTRs), some as long as 10 kb, and exhibits the greatest frequency of APA. 3’ UTR lengthening occurs as neural progenitor cells differentiate into post-mitotic maturing neurons, exposing additional 3’ UTR regulatory elements such as RBP targets supporting mRNA trafficking [12]. Although neural tissues do not broadly show similar lengthening of 5’ UTRs, brain-expressed transcripts rely heavily on alternative promoter usage to generate isoform diversity, resulting in distinct transcription start sites (TSS). Additionally, these transcripts tend to have more ‘complex’ 5’ UTRs, for example, more frequently harboring upstream open reading frames (ORFs) and conserved noncoding sequence elements [13].

Given the notable transcriptomic complexity in the brain, it is perhaps unsurprising that RNA processing and co-transcriptional regulation have increasingly been implicated as NPD risk mechanisms. Tremendous progress has been made over the past decade in identifying hundreds of high-confidence NPD risk genes/loci via large-scale genome-wide association (GWAS) and whole-exome sequencing (WES) studies [14,15]. Despite this progress, functional mechanistic interpretation has remained difficult [14,16,17]. GWAS hits are predominantly noncoding and often located within large blocks of linkage disequilibrium, obscuring the true underlying ‘causal’ variant(s) and target gene. In contrast, WES requires accurate prediction of a variant’s impact on protein coding sequence, resulting in many variants of uncertain significance (VUS) and many genetically suspected cases remaining undiagnosed. Both approaches are typically interpreted at the gene level, which can miss regulatory variants that shift isoform usage. Growing evidence shows that disease-associated variants frequently alter splicing or isoform expression rather than total gene expression [4–6,14]. Brain region- and cell type-specific isoform profiles can help interpret patient VUS [18], and disruptions of brain-expressed RBPs and spliceosomes cause NDDs [12,19]. For example, pathogenic variants in spliceosomal snRNA genes such as *RNU2–2* and *RNU4–2* cause severe developmental phenotypes [20,21]. Consistent with this, dosage-sensitive genes associated with developmental disorders exhibit increased length and structural complexity of 5’ UTRs [13]. Alternative TSS usage has also recently been shown to be tightly coupled with 3’ end site choice, in a tissue-specific manner, directly linking 5’ and 3’ regulation of isoform selection [22]. Collectively, these findings underscore the central role of isoform-level regulation in human brain biology.

## Long read sequencing: an expanded view of isoform diversity in human brain

**Figure 2:**
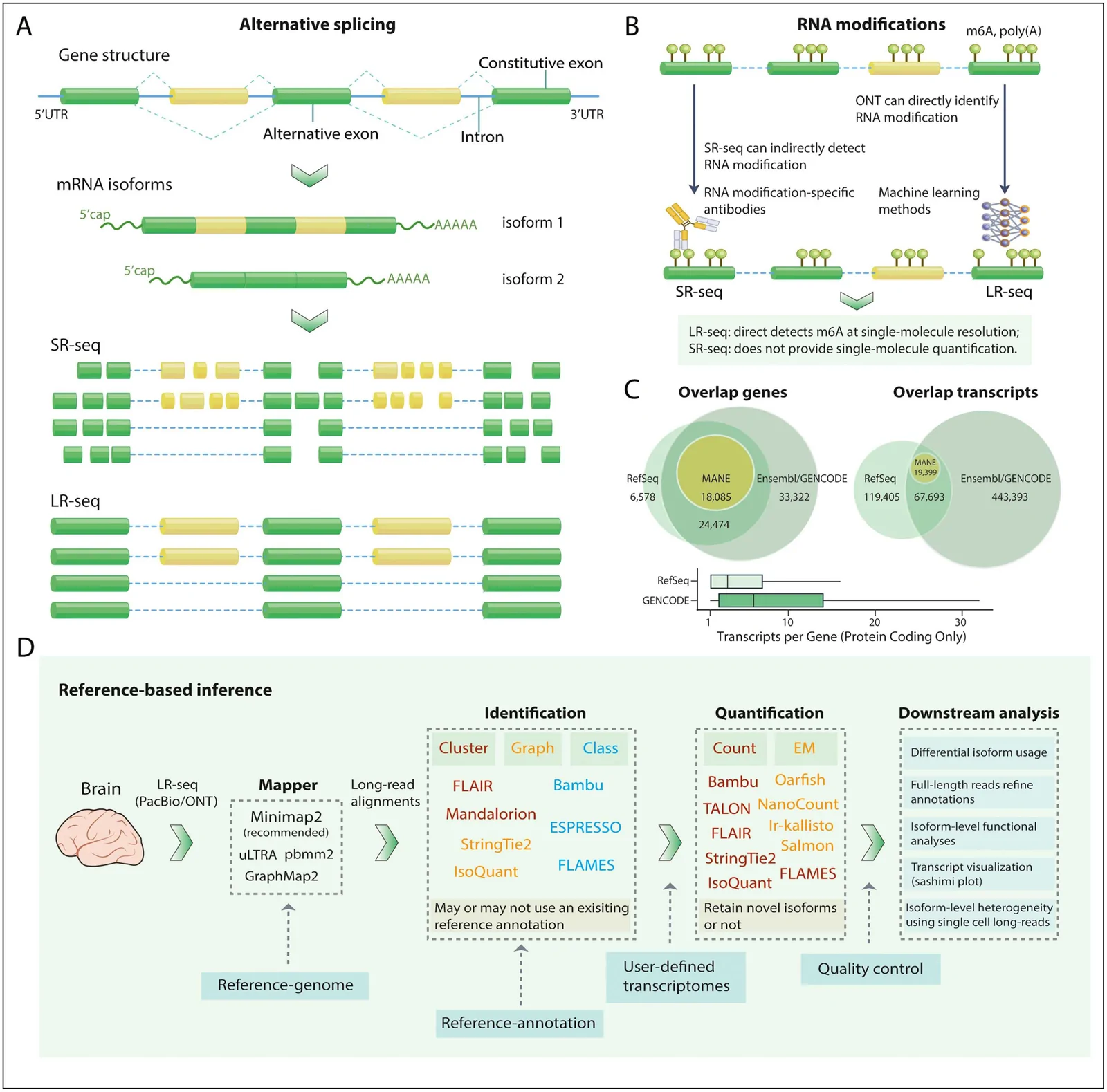
Overview of long-read sequencing applications and reference-based transcript quantification and identification. **(a)** Alternative splicing in SR-seq vs LR-seq. Schematic diagram of an alternatively spliced gene producing two isoforms. Short-read RNA-seq (SR-seq) quantifies exons and splice junctions with high precision, but because reads span limited distances, long-range exon phasing is ambiguous, and full-length isoforms often require inference. Long-read RNA-seq (LR-seq) preserves exon continuity within single molecules, directly resolving full-length isoforms, complex splicing patterns, and highly similar transcripts; specialized alignment and isoform-collapsing/error-handling are typically required, and platform-specific biases should be considered. **(b)** RNA modifications in SR-seq vs LR-seq. RNA modifications (e.g. m6A) regulate splicing, nuclear export, translation, and RBP recruitment in the brain. SR-seq approaches detect modifications indirectly (antibody-based enrichment, chemical/enzymatic treatments, or RT-signature-based inference), providing population-level estimates without single-molecule quantification. In contrast, ONT direct RNA LR-seq interrogates native RNA and uses raw-signal features with machine-learning models to call modifications per read, enabling base-level localization. **(c)** Overlap of genes and transcripts across major reference annotation databases. Venn diagrams depict the overlap in the number of genes (left) and transcripts (right) among GENCODE, Ensembl, RefSeq, and the MANE reference set. Numbers indicate unique and shared subsets across these annotation resources. Boxplots (bottom) illustrate the distribution of protein-coding transcript counts per gene in RefSeq and GENCODE annotations. **(d)** Reference-based transcript identification and quantification using LR-seq data. Long reads (PacBio/ONT) are mapped to a reference genome (minimap2 recommended). Transcripts are identified via clustering/graph/classification approaches (e.g. FLAIR, StringTie2, IsoQuant, Bambu, ESPRESSO, FLAMES) with or without existing annotations. Quantification can be count-based (Bambu, TALON, FLAIR, StringTie2, IsoQuant, FLAMES) or expectation-maximization (EM)-based (Oarfish, NanoCount, lr-kallisto, Salmon). After quality control, quantification results can be used for downstream analyses.

The advent of high-throughput short-read RNA-sequencing (SR-seq, circa ~2008) transformed our understanding of the transcriptome, enabling unbiased discovery and quantification of expressed transcripts genome-wide, along with patterns of AS, polyadenylation, and TSS usage. However, SR-seq is inherently limited in its ability to reconstruct full-length transcript-isoforms, particularly for long genes with complex isoform structures, such as those enriched in the brain (Figure 2a). Consequently, it is increasingly recognized that existing gold standard genome annotation databases are incomplete, and most SR-seq analyses underappreciate the regulatory roles of 5’ and 3’ UTRs [1]. Technological advances from PacBio and Oxford Nanopore (ONT) enabling long-read RNA sequencing (LR-seq) with high throughout and fidelity now enable scalable profiling of individual full-length transcripts from bulk tissue or single cells without assembly, resolving isoforms and fine distinctions (alternative TSSs, poly(A) sites, exon usage), improving mapping in repeat-rich regions, and detecting RNA base modifications (Figure 2b). Demonstrating scalability, LR-seq profiling was conducted across 88 GTEx tissues and cell lines, uncovering 71,735 previously unannotated transcript-isoforms of known genes [3]. Beyond new isoforms, LR-seq has also revealed many new genes — particularly long noncoding RNA genes — highlighting the incompleteness of reference catalogs including GENCODE, ENSEMBL, and RefSeq. For instance, the gold standard GENCODE reference added > 15k new human genes from 5/2024 (v46) to 9/2025 (v49; current release), with the number of annotated transcripts nearly doubling, from 254,070 to 507,365 [23]. Furthermore, only modest overlap exists between RefSeq and GENCODE/ENSEMBL (Figure 2c), underscoring the need for both more comprehensive and harmonized isoform annotations.

Many of the largest gains in transcript-isoform discovery with LR-seq have come from profiling of the mammalian brain, particularly during human neurodevelopment, as these tissues and timepoints have been among the least accessible for large-scale transcriptomics [9,12,24]. Bulk LR-seq in mouse and human brain revealed widespread transcript diversity, with many isoforms missed by SR-seq [2,9,10]. To minimize interindividual confounders, one study profiled cerebellum, hypothalamus, and temporal cortex from the same donors [10], identifying multiregion isoforms and region-dependent isoform usage in genes. A large proportion of novel transcripts were full-length, exhibiting unique intronic splicing patterns. Consistent with prior work, these tended to be longer with more exons than annotated isoforms [9]. Genes whose dominant isoform shifted across regions were enriched for dendritic morphology and neuronal pathways, suggesting region-specific functional programs. Developmental regulation is also prominent, with differential transcript usage separating developing from adult cortex [9] and distinguishing the progenitor-rich germinal zone (GZ) from the post-mitotic cortical plate (CP) in the developing neocortex at mid-gestation [12].

More recently, single-cell and spatial LR-seq (scLR-seq) extensions have been developed, enabling high-resolution mapping of cell type- and region-specific transcript usage across mouse, human, and organoid brains [8,12,18,19,25–27]. In mouse hippocampus and prefrontal cortex, scLR-seq detected hundreds of genes exhibiting cell-type-specific differential isoform usage, including cases where regional identity overrode cell-type specificity (e.g. *Nsfl1c* restricted to hippocampus) [19,28]. scISOr-Seq revealed coordinated spatial isoform regulation [19,28], while SnISOr-Seq increased barcoded exon-spanning reads 7.5-fold and exposed highly cell-type-specific exon inclusion in adult frontal cortex [18]. The improved ScISOr-Seq2 expanded profiling across regions, cell types, development, and species; 72% of genes showed full-length isoform variation, with regulation especially dynamic during adolescence [8,27]. Complementary bulk and single-cell LR-seq of GZ and CP uncovered thousands of neurogenesis-associated isoform switches, most with observed or predicted functional effects; 57% were predicted to alter ORF length [12]. Isoform-level resolution sharpened cell-type-specific co-expression network assignment, highlighted early excitatory neurons as harboring the greatest isoform diversity, and revealed previously uncharacterized cell states, consistent with isoform-defined classifications [12,19]. Together, LR-seq — especially at single-cell and region-resolved scales — overcomes SR-seq limitations to reveal extensive, cell-type- and development-specific isoform complexity in the human brain.

## Quantification and functional annotation of transcript isoforms

Despite substantial recent methodologic progress [1], major technical and computational challenges continue to impede accurate isoform identification and quantification from LR-seq data. Methods must distinguish true novel biology from technical or methodological artifacts, which can be introduced at multiple stages across library construction, sequencing, and analysis. Notably, ONT and PacBio platforms exhibit characteristic differences in technical biases, with distinct error rates, base composition, and length distributions. Sequencing biases are often magnified with single-cell approaches, particularly in the human brain, which generally requires nuclear isolation, and often exhibits more RNA degradation than other, more accessible tissues. Additional sources of technical biases commonly arise from polymerase chain reaction amplification, reverse transcriptase-mediated template switching, internal priming, and sequencing fidelity of longer molecules, among others, which can collectively skew downstream analyses [29,30]. Notably, whereas RNA degradation results in a characteristic 3’ read coverage bias, internal priming — particularly prominent with nuclear RNAs — introduces a 5’ bias. Although some of these biases are addressed by current LR-seq analysis methods, such as by filtering out reads with evidence of internal priming [30,31], this can discard a large fraction of raw reads, and no single method currently models all major sources of technical artifacts.

Even with high-quality input data, LR-seq quantification methods are impeded by the lack of a ground-truth transcriptome annotation. Multiple methods support reference-guided or *de novo* transcriptome assembly and quantification [30–33], yet results often diverge substantially across approaches, even when applied to the same dataset [29]. Algorithms can be broadly categorized into class-based, graph-based, and cluster-based strategies, and can also be distinguished by their degree of reliance on external reference information, ranging from annotation-guided to genome-guided but annotation-free, or fully reference-free. Annotation-guided methods use existing transcript annotations to map, or pseudo-align reads to a reference transcriptome, providing efficient quantification but potentially biasing results toward known isoforms (Figure 2d). Examples include class-based ESPRESSO [34] and Bambu [31]; graph-based StringTie2 [35] and IsoQuant [30]; and cluster-based Mandalorion [36] and FLAIR [37]. While many of these tools support annotation-guided quantification of known isoforms, several (e.g. Bambu, IsoQuant, StringTie2) also enable discovery of novel isoforms in annotation-independent settings.

Genome-guided, annotation-free approaches reconstruct isoforms using only the reference genome, avoiding transcript annotation bias, and enabling novel isoform discovery, albeit at increased computational cost and with greater dependence on accurate splice-junction detection. Annotation-free tools such as Freddie [38] detect and quantify isoforms without relying on transcript annotations. Fully reference-free methods cluster reads without using either genome or transcript annotations, making them applicable to poorly annotated organisms, though with reduced precision in highly complex splicing contexts; for example, RNA-Bloom2 [39] and RATTLE [40] infer splice variants solely from reads clustering when neither reference genomes nor annotations are available. Direct quantification methods (e.g. Oarfish [41], and LIQA [42]) explicitly model read assignment and technical bias to improve isoform abundance estimates, but require a known reference. Finally, hybrid approaches are being used to integrate within-sample LR-seq and SR-seq profiling, for instance, using LR-seq to construct a sample-specific isoform reference while leveraging higher depth of SR-seq for quantification [43].

Several systematic benchmarking studies have recently been conducted, prioritizing Bambu and IsoQuant for sample-specific transcriptomes in well-annotated organisms; Mandalorion and FLAIR (with short-read support) for rare/lowly expressed isoforms; LyRic for well-supported novel transcripts; and IsoQuant, FLAIR, and Bambu for quantification [29,44]. Given the technical biases and methodological challenges outlined above, as LR-seq continues to uncover novel unannotated isoforms, typically of low abundance, such transcripts should be assessed for orthogonal supporting evidence [1]. For instance, ORF prediction and proteogenomic validation can provide support for protein-coding transcripts, whereas noncoding genes/transcripts can be evaluated for evolutionary conservation. Finally, although it may be tempting to ignore lowly expressed and/or non-coding transcripts (e.g. NMD-inducing) of protein-coding genes, we note that even such rare splicing events can have substantial clinical relevance for variant interpretation in suspected Mendelian disorders. Strengthening the robustness and reproducibility of isoform quantification is therefore critical for leveraging LR-seq in human brain transcriptomes.

This challenge becomes even more acute in scLR-seq, where isoforms of potential mechanistic interest — such as cell-type-restricted minor isoforms, developmentally regulated isoform switches, or microexon-containing neuronal transcripts — may be inefficiently captured due to the sparsity of single-nucleus/cell coverage. Several complementary strategies can mitigate this limitation. Physical enrichment of defined cell populations, through fluorescence-activated cell sorting, magnetic-activated cell sorting, or genetically labeled cells, followed by high-depth ‘bulk’ LR-seq, is a sensitive approach, as recently illustrated [24]. However, this comes at the cost of lower throughput and cell-state granularity. Second, target-capture-based approaches, including TEQUILA-seq [45] and IsoLamp [46], can selectively increase coverage over disease-relevant loci, enabling detection of cell-type-specific splicing changes, previously uncharacterized isoforms, and novel exons that may be missed in transcriptome-wide or bulk analyses. Third, improved sequencing and quantification strategies can increase confidence in low-abundance transcript detection: higher-throughput long-read chemistries, such as PacBio MAS/Kinnex, increase long-read yield, whereas accuracy-focused approaches, such as ONT duplex sequencing, improve read-level confidence; in parallel, hybrid short–long-read quantification frameworks, including Bambu [31], IsoQuant [33], lr-kallisto [47], and Oarfish [41], can improve the sensitivity to detect and quantify low-abundance transcripts. Finally, single-cell aggregation-based analyses (e.g. pseudo-bulk or metacell) across transcriptionally homogeneous single-cell clusters can restore detection power while retaining cell-type-level resolution. Together, these approaches define a practical design space for isoform-resolved single-cell studies of the human brain, balancing sensitivity, throughput, and cellular resolution.

## Genetic regulation of isoform diversity in human brain

**Figure 3:**
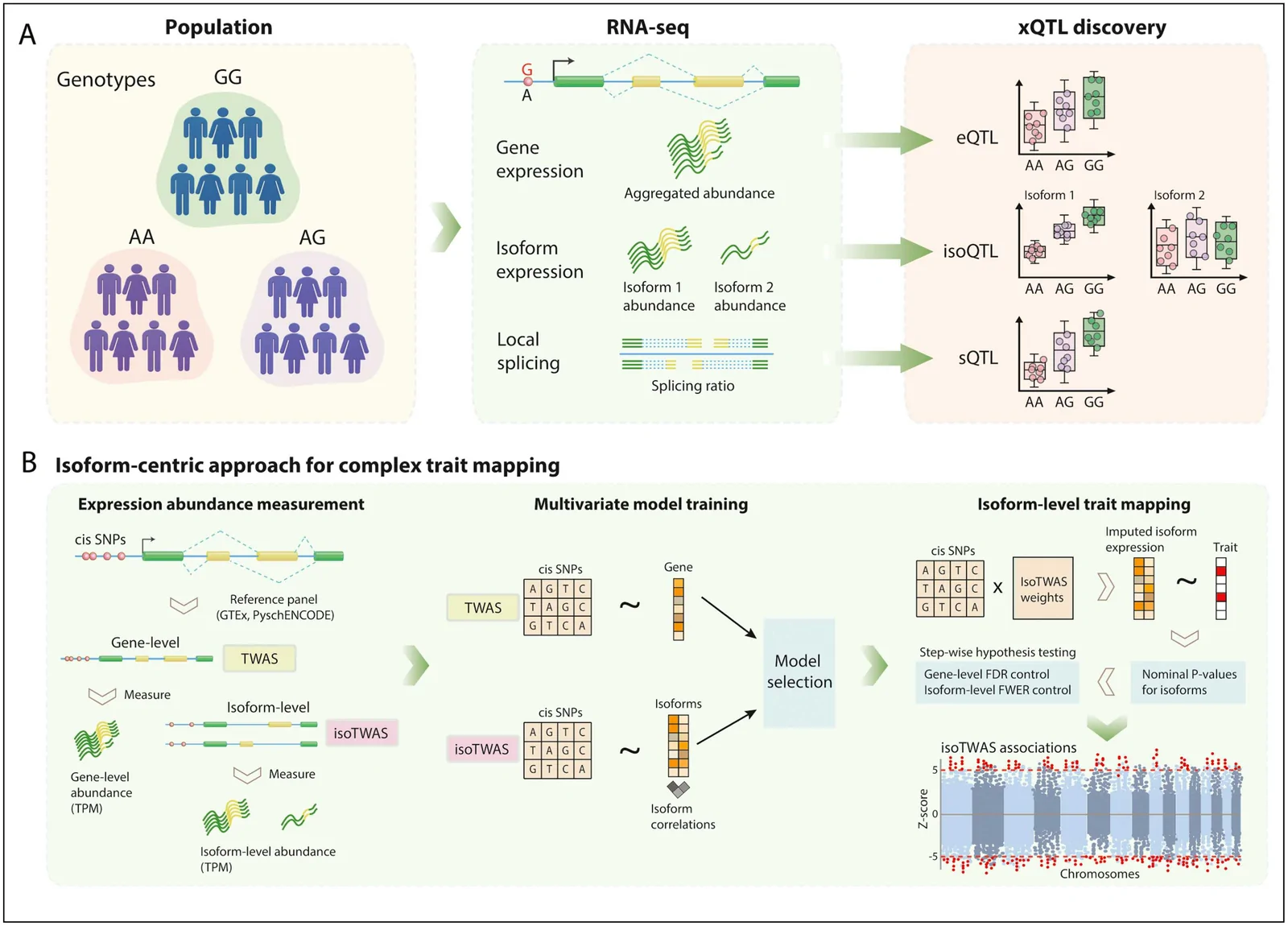
From population xQTL discovery to isoform-centric trait mapping. **(a)** xQTL discovery across molecular readouts. Individuals are grouped by *cis* SNP genotypes (e.g. AA, AG, GG). RNA-seq enables three complementary molecular phenotypes: (i) gene expression (aggregate abundance across isoforms), (ii) isoform expression (abundances of individual isoforms), and (iii) local splicing (junction/percent-spliced-in ratios). Association testing identifies eQTLs, isoQTLs, and sQTLs, illustrated as genotype-stratified effect distributions. **(b)** Isoform-centric framework for complex-trait mapping (isoTWAS). Using functional-genomics reference panels (e.g. GTEx, PsychENCODE), isoTWAS trains multivariate models to predict isoform expression from *cis*-window SNPs, whereas gene-level TWAS fits univariate models for total expression. Models with cross-validated R^2^ > 0.01 are retained, and their weights are combined with GWAS summary statistics to impute isoform expression and test trait associations. A stepwise testing framework provides gene-level false discovery rate (FDR) control and isoform-level family-wide error rate (FWER) control; locus-level permutations account for local LD and GWAS architecture, with optional Bayesian fine-mapping when multiple signals occur. Genome-wide signed Manhattan plot of isoTWAS Z-scores based on tissue-specific isoQTL weights (e.g. developing brain) integrated with GWAS (e.g. SCZ or ASD). Each dot represents an isoform; isoforms with adjusted P < 0.05 are highlighted in red.

Genetic variation can shape isoform diversity by modulating promoter and TSS usage, local splicing events within a transcript, as well as APA — regulatory processes, all prevalent in the human brain and implicated in NPDs. Splicing quantitative trait loci (sQTLs) characterize variant effects on alternative local splicing regulation, such as exon inclusion, splice-junction usage, or intron retention, each representing individual steps within the broader splicing process [48]. sQTLs have been shown to represent a critical mechanism linking genetic variation with human disease, largely orthogonal to gene expression regulation [4,49]. In contrast, expression QTLs (eQTLs) map variants that influence total gene-level abundance regardless of isoform composition. However, transcriptional regulation ultimately operates at the isoform level, as individual mRNA transcripts can differ in promoter choice, splicing-junction and exon usage, intron retention, and polyA site selection — each susceptible to modulation by *cis*-acting genetic variants. Isoform QTLs (isoQTLs) therefore extend beyond eQTLs and sQTLs by linking genetic variants directly to full-length transcripts, capturing the combined regulatory consequences of promoter usage, splicing, and 3’ end processing that cannot be fully resolved by analyses of gene-level or local splicing analyses alone (Figure 3a).

Several studies have conducted sQTL profiling in the developing and adult human brain using bulk SR-seq, observing that sQTLs are important and independent contributors to disease risk [14,17,48]. Large-scale human-cortex datasets reveal widespread genetic control of splicing with sQTLs implicated across schizophrenia (SCZ), ASD, and bipolar disorders (BIP), and many effects are developmentally stage-specific [5,6,14,17,50]. Methodologically, sQTL mapping has been performed at two distinct levels. Transcript-level methods (e.g. THISTLE [17] and sQTLseekeR [51]) estimate relative isoform abundance by aligning (short) reads to annotated transcripts such as GENCODE. Event-level methods (e.g. LeafCutter [52] and MAJIQ [53]) estimate local splicing event inclusion (percent spliced in, PSI) by quantifying reads over exons or splice junctions. Because they do not rely on transcript annotations, they can capture novel splicing changes — particularly relevant in the brain. However, because SR-seq rarely resolves full-length structures, most eQTL/sQTL analyses offer exon- or junction-level surrogates rather than direct isoform-resolved effects.

isoQTLs more directly link genetic variants to full-length transcript isoforms, offering a more integrative view of post-transcriptional regulation (Figure 3a). Beyond the harmonization challenges of local events, isoform abundances can be estimated across cohorts using scalable pseudoalignment. In the brain, isoform-level expression changes show stronger enrichment of SCZ SNP-heritability than gene-level or local splicing changes [4,6,14]. Furthermore, isoQTLs in the developing human brain were found to mediate more SNP-heritability across several NPDs than at gene or splicing levels [14]. While isoQTL discovery has been previously limited by short reads, large-scale population-level LR-seq profiling studies are beginning to address this constraint, revealing regulatory signals missed by conventional gene-level frameworks and showing notable cell-type and region specificity [3,24].

Moving from QTLs to trait mapping, transcriptome-wide association studies (TWAS) integrate population-scale transcriptomic reference panels with GWAS summary statistics to prioritize putative underlying gene-to-trait associations. Traditional TWAS approaches, however, model gene- or local splicing regulation, thereby obscuring potential isoform-specific genetic effects. To address this limitation, isoTWAS developed a multivariate framework to jointly model SNP effects on all expressed isoforms of a given gene (Figure 3b), yielding substantial gains in discovery power [4]. Across 15 NPDs, isoTWAS increased detection of trait-associated isoforms by ~40% and identified associations at ~60% more GWAS loci relative to conventional TWASs [4]. Consequently, isoform-resolved genetic analyses can provide a critical mechanistic bridge between regulatory variation and neuropsychiatric disease etiology that cannot be captured at the gene level alone. Of note, current isoTWAS models are trained on short-read RNA-seq-derived isoform abundances quantified by pseudoalignment against existing annotations, and therefore share limitations of genome-guided, short-read sequencing: they cannot detect unannotated isoforms and have limited ability to resolve distal splice-event phasing within long, complex brain-expressed genes. Retraining isoTWAS on emerging bulk and single-cell long-read reference panels of the developing, adult, and cell-type-resolved human brain is therefore an important next step. Complementary to isoTWAS, SpliTWAS [54] extends TWAS to exon-level splicing by modeling genetically regulated inclusion values, followed by integration with GWAS summary statistics and by probabilistic fine-mapping. Together, gene-level TWAS, exon-level SpliTWAS, and isoform-level isoTWAS provide a complementary hierarchy for linking regulatory genetic variation to neuropsychiatric disease through post-transcriptional regulation.

## Widespread isoform diversity in genes implicated in brain disorders

Genes implicated in NPDs often display complex isoform usage, pointing to isoform dysregulation as a mechanistic axis of disease pathogenesis [2,9,12,25,27]. A curated analysis of ASD, SCZ, and Alzheimer’s disease (AD) genes found that most express multiple RNA isoforms in human (68.9%) and mouse cortex (80.3%); notably, 39.2% of human and 56.7% of mouse isoforms were unannotated, underscoring the potential disease relevance of transcript diversity not captured by existing annotations [9]. LR-seq further revealed that ASD-associated exons exhibit the greatest cell-type-specific inclusion variability among exons differentially spliced in ASD, SCZ, and amyotrophic lateral sclerosis (ALS), thereby linking disease-associated splicing to specific cellular contexts [25]. Consistently, ASD brains show exon inclusion profiles that resemble progenitor states more than mature neurons [25], and ASD proband *de novo* mutations (DNMs) are enriched near exon junctions — particularly within cell type-specific exons — as predicted by SpliceAI, a deep learning model trained to detect cryptic splice mutations from pre-mRNA sequences [55]. Moreover, 76.8% of ASD-associated genes are differentially spliced across neural cell types, reinforcing the centrality of isoform-level regulation [25].

Rare variant studies extend these findings. WES/WGS analyses of ASD, NDD, and developmental disabilities (DDD) genes show that disease-associated genes harbor more isoforms and exons than nondisease genes, overlap extensively with genes dynamically expressed, translated, and spliced during cortical neurogenesis, and are enriched in isoforms in excitatory neurons [12]. Co-expression networks further localize disease signals primarily to isoform-expression modules, followed by isoform-usage and then gene-expression modules, with enrichment for cytoskeleton regulation, synaptic vesicles, neurite morphogenesis, and ribosomal RNA processing and chromatin regulation — consistent with previous findings [6]. Complementing exon-level findings, isoform-centric analyses have systematically reprioritized DNMs at the transcript level. An isoform-focused atlas [12] mapped thousands of DNMs linked to ASD, intellectual disability, and NDDs to specific isoforms, uncovering a higher burden of cryptic splice variants than previously reported. Extending beyond developmental stages, deep LR-seq of aged human frontal cortex identified 1917 medically relevant genes expressing multiple isoforms, including 1018 with distinct protein-coding sequences and 57 linked to brain disorders such as SCZ and major depression [2]. Remarkably, 53 isoforms were previously unannotated, several representing the predominant transcript for their gene. Altogether, these findings highlight that brain disease-associated genes harbor widespread, functionally important isoform diversity and that isoform number and complexity are strong predictors of disease relevance.

Beyond expanding the catalog of disease-associated transcripts, isoform-centric analyses have begun to yield concrete mechanistic and cellular insights into human brain disorders. In idiopathic ASD, reduced inclusion of a neuron-specific 24-nt microexon in CPEB4 shifts this RBP from dynamic condensates toward solid-like aggregates and is associated with widespread poly(A)-tail shortening among ASD-risk gene transcripts, providing a direct isoform-to-pathology link that is not evident from gene-level expression alone [11]. At the cellular level, full-length isoform profiling of developing human neocortex and ASD-related models refines neuronal subtype annotation beyond gene-level scRNA-seq, resolving previously underappreciated excitatory-neuron states and revealing neurogenesis-linked isoform switches that alter protein domains and RNA-regulatory elements [12,25]. These examples illustrate that moving from gene-level to isoform-level analysis is not simply a technical refinement, but a means to uncover disease mechanisms, disease-relevant cell states, and candidate therapeutic targets that are otherwise obscured.

## Conclusion

Transcript isoform diversity constitutes a pervasive, functionally consequential layer of gene regulation in the brain. Advances in long-read and single-cell technologies now enable cell-type- and region-resolved reconstruction of full-length isoforms, uncovering thousands of previously unannotated transcripts and delineating neurogenesis-linked and disease-associated splicing programs. Isoform-centric analyses reveal that genes implicated in NPDs exhibit markedly greater splicing complexity and harbor an excess of *de novo* and cryptic splice mutations relative to nondisease genes. Integrating isoform-resolved expression into variant interpretation frameworks will be critical for clarifying the mechanistic basis of disease and improving diagnostic precision. Looking ahead, multimodal approaches integrating long-read, spatial, and proteogenomic datasets with robust quantification and functional assays will prioritize causal isoforms and sharpen mechanistic understanding of brain disorders.

## Data Availability

No data were used for the research described in the article.
